# Fatigue Due to Multiple Sclerosis: A Comparison Between Persian Medicine and Conventional Medicine

**DOI:** 10.31661/gmj.v8i0.1139

**Published:** 2019-05-09

**Authors:** Hossein Rezaeizadeh, Roja Rahimi, Maryam Abbasi

**Affiliations:** ^1^Department of Traditional Iranian Medicine, School of Traditional Medicine, Tehran University of Medical Sciences, Tehran, Iran; ^2^Department of Traditional Pharmacy, School of Traditional Medicine, Tehran University of Medical Sciences, Tehran, Iran

**Keywords:** Fatigue, Medicinal Herbs, Multiple Sclerosis, Medicinal Plants, Traditional Medicine

## Abstract

Fatigue is one of the most debilitating symptoms of multiple sclerosis (MS), and its definite pathophysiology is unclear. Studies have suggested some correlates for it including dysfunction or atrophy in different parts of the brain. This narrative review study compares the viewpoint of conventional medicine and Persian medicine (PM) about fatigue due to MS and introduces the treatments used for this complaint in PM with an evidence-based approach. PM scholars have used the term *I’ya* equal to fatigue and stated that* I’ya* might be due to exertion or not, while the latter (spontaneous *I’ya*) can be prodromal of a disease. This pathologic fatigue can be seen in a wide variety of neurologic diseases, though it is the most common in MS patients. Fatigue in MS can be considered one of the equivalents of spontaneous* I’ya*. According to PM texts, neurotonic herbs like *Ferula*, *Citrus medica*, *Asarum europaeum*, *Ficus carica*, and *Juglans regia* may be beneficial in alleviating fatigue by brain reinforcement. Different pharmacological mechanisms have been introduced for these plants including antioxidant and/or anti-inflammatory activities. The medicinal plants can be assumed as a valuable source for discovering new medicines for fatigue in MS. Designing preclinical and clinical studies evaluating the effects of mentioned medicinal herbs in fatigue is proposed for obtaining more conclusive results.

## Introduction

Fatigue is defined as a subjective lack of physical and/or mental energy that is perceived by the individual or caregiver to interfere with usual or desired activity [1]. It is a common annoying symptom in many chronic illnesses especially neurologic diseases. In both central and peripheral nervous system (CNS, PNS) diseases, fatigue may occur in certain periods of disease, but causal mechanisms may differ. It seems that the frequency of fatigue in multiple sclerosis (MS) is among the highest compared to other chronic diseases and is one of the most important factors for deterioration of quality of life in MS patients [2, 3]. Fatigue in MS is a complex event with neither consensus about its definition, nor gold standard for measuring. Furthermore, its origin and the exact underlying mechanisms are still unknown [4]. In the present study, fatigue has been investigated in Persian medicine (PM) literatures from different aspects including types, etiologies, and similarities to MS fatigue. Moreover, medicinal plants introduced for this complaint in PM have been reviewed, and possible pharmacological activities related to their efficacy in this issue have been discussed. Also, the fatigue of MS has been investigated in the literature of conventional medicine searching for its underlying factors and comparison with PM theories. To the best of our knowledge, no review study was found about fatigue comparing conventional and traditional medicine. It seems that the utilization of therapeutic options of traditional and alternative medicine may have some benefits in this case.

## Search Strategies

This narrative review about fatigue in MS and its equivalent in PM aiming to find potential remedies in PM. Electronic databases including PubMed, Scopus, and Google Scholar were searched for any articles about fatigue in MS including reliable definition, suggested etiologies, classifications, and correlations. Articles were searched from January 1995 till May 2017. Reference lists of retrieved articles were manually reviewed also to find additional applicable studies. The search terms were: “fatigue,” AND “multiple sclerosis” OR “MS.” To find relevant studies, primary search results were screened through reading titles and abstracts. The selected articles were then checked by their full text and finally included articles were determined. Besides, PM manuscripts including The Canon of medicine, *Al-havi fi tibb*, *KholasatOl- Hikmah* and *Ibn Rushd’s* main book (*Kulliyat* or *Colliget*) were scrutinized for any synonyms of fatigue, the assortment, causes of fatigue and the available remedies in PM. In old PM literature, some herbs were found that had been introduced for alleviating fatigue. Further on researches were done on the functions and effects of these herbs on the nervous system in mentioned electronic databases. The search terms for this part were the scientific name OR common name of each herb in English, with fatigue, OR multiple sclerosis, OR lassitude, OR exhaustion, OR tiredness, OR nervous system, OR neuroprotection, OR brian tonics, OR neurotonic.

### Fatigue in PM Manuscripts

The term, *I’ya* in PM texts has been considered as fatigue [5] or lassitude [6] and is defined as excessive fatigue or weakness that hinders or diminishes working ability. PM scholars state that *I’ya* may be caused by exertion or not, and the latter (spontaneous fatigue) [7] can be a prodromal phase of the disease [8-10]. PM scientists have indicated that spontaneous *I’ya* usually results from the accumulation of waste materials in the body. The matters in the body should pass a rheological change – named *Nozj* phenomenon – to be ready for consumption or discretion [11]. If the waste matters cannot be excreted from the body completely, due to their inappropriate consistencies or failure of exit pathways, they accumulate in different parts of the body which in turn generate different signs and symptoms such as *I’ya*. Depending on the type of accumulated waste matters in the body, the accompanying symptoms of *I’ya* can differ. PM scholars have divided *I’ya* to three different types [7, 9]: *I’yaol-qoruhii, I’yaol-tamadodii*, and *I’yaol-varamii.* In PM school, the human body is formed from four types of fluids (humor or *Akhlat*) [12]: phlegma, blood, black bile and yellow bile[13]. The balance of these humors with each other is essential for health, and either excess or diminution of one of them can lead to disease. PM scholars consider spontaneous *I’ya* secondary to the surplus of these humors. *Ibn Rushd* ( *Averroes*) one of the PM scientists in the 12th century, has stated that it is possible that the waste matters are not excessive in fact, but the body is not capable of bearing them due to weakness in one or several essential organs (liver, heart, and brain) [14]. It means that the weak organ cannot handle even this moderate amount. *Avicenna* emphasizes that weakness of any organ can be caused by congenital or acquired factors. He points out chronic or severe illness in the organ as one of the causes of organ weakness [9]. From this perspective, the spontaneous *I’ya* can be considered equivalent to the pathologic fatigue which is seen in chronic diseases especially MS. Although MS has not been described as a distinct disease in PM texts, most of its prominent symptoms and signs such as paresthesia/hypoesthesia, limb paresis, and plegia have been well explained as separate disease s[15]. *Hally Abbas* and *Ibn Rushd*, two of famous PM physicians, have described a disease that begins with optic nerve inflammation/obstruction and may lead to paresthesia or hypoesthesia [15], which resembles the course of MS in many affected patients.

### Classification of Fatigue in Conventional Medicine

Generally physiologic fatigue after activities in healthy individuals is acute and transient which mostly resolves by rest [16] and emanates from both PNS and CNS [17], while chronic fatigue that is related to medical illnesses occurs regardless to activities, it usually lasts more than six months, rarely relieves after rest and could be multifactorial [16]. Different terms for it is used in the articles including mental fatigue, cognitive fatigue, motor fatigue, physical fatigue, lack of motivation or energy, worsening of symptoms, tiredness, asthenia, fatigability, and lassitude[4]. Fatigue in MS patients may be primary or secondary [18]. Some etiologies have been suggested and examined for primary fatigue in MS, such as pro-inflammatory cytokines, neuroendocrine factors, brain lesions (plaques), axonal damages and functional changes in cortical activation [4, 18], although controversy was found in several articles about the correlation of these factors with fatigue. Secondary fatigue is attributed to sleep disturbances, pain, disability, depression, and other psychological factors and medication side effects in MS patients [4, 18, 19]. From another point of view, fatigue can be classified to peripheral and central fatigue. Peripheral fatigue is more definable and measurable, unlike central that is more complex and hardly assessable [20]. Both central and peripheral fatigues are seen in MS nevertheless it seems that central fatigue is mainly responsible [21].

#### 1. Central Fatigue

There are several definitions about central fatigue at present; the main characteristic of central fatigue is increased perceived effort and reduced endurance to sustain motor or cognitive activities [22]. Based on the neurophysiological assessment, some articles have found that pre-movement facilitation is missing in fatigued MS patients, which correlates with fatigue grade. They suggested that dysfunction in motor areas of the brain cortex leads to loss of pre-movement facilitation and consequently earlier fatigue [23]. Some changes in serotonergic transmissions in some parts of the brain including limbic and paralimbic and also frontal cortex in MS patients were found having a correlation with fatigue and depression in them [24].

#### 2. Peripheral Fatigue and Fatigability

Peripheral fatigue has been defined as the inability to maintain force during muscle contraction and most often is seen in PNS diseases or neuromuscular junction disorders such as myasthenia [22]. There are also two distinct entities about fatigue; asthenia (or fatigue in a rest state and without activity) and fatigability (fatigue with mild exertion) [4]. One study indicated a correlation between asthenia and immunoactivity as well as fatigability and pyramidal involvement in MS patients. In this study, 72% of the patients described their fatigue as fatigability and 22%, as asthenia [25]. Another study found higher levels of pro-inflammatory cytokines in fatigued-MS patients versus not fatigued. Also, they showed a positive correlation between tumor necrosis factor-α levels and daytime sleepiness in patients [26]. Nevertheless, the evidence for proving the role of inflammatory factors in MS fatigue is not sufficient yet [27].

### Anatomical Brain Correlates of Fatigue

Several researchers have attempted to discover possible correlation of MS fatigue with some parts of the brain, using magnetic resonance imaging (MRI) [23, 28-33], functional MRI (fMRI) [34-36], MRI spectroscopy [23, 37, 38], transcranial magnetic stimulation [23], positron emission tomography [39] or electroencephalography [40]. Several studies have indicated an abnormality of gray matter including deep and/or cortical gray matter in fatigued MS patients. Basal ganglia and striatothalamocortical (frontal) system dysfunctions are suspected to have an essential role in fatigue due to MS [22, 29, 34, 35, 38, 39, 41]. The evidences for the role of different parts of the brain in MS fatigue are displayed in Table-1. There is controversy in literatures about the correlation of white matter lesion loads (burden of the plaques) and fatigue [23, 32, 33].

### Medicinal Plants for Fatigue According to PM

Several medicinal plants were introduced in PM literatures for amplification of the nervous system and management of fatigue. Some of these herbs as well as current scientific evidences that confirm their effectiveness for this purpose have been discussed below.

#### 1. Ferulaassa-foetida

*F. assa-foetida* oleo-gum-resin that called “*Heltit*” in PM is one of the herbal samples that used for reinforcement of the brain and management of fatigue. In a study on neuronal cells cultured from adult rats, *F. assa-foetida* aqueous extracts in certain concentrations, increased survival rates of neurons [42]. In another study, *F. assa-foetida* extracts had a neuroprotective effect in cerebellar neurons of rat and reduced glutamate-induced cell death via apoptosis or necrosis [43]. One of the active constituents of *ferula* oleo-gum-resin is ferulic acid (FA). FA had neuroprotective effects via stimulating nerve regeneration in some animal studies [44]. Also, it reduced apoptosis due to oxidative stress in a murine model of cerebral ischemia by inhibiting intercellular adhesion molecule – 1 expression [45]. There are some other components in *ferula* such as flavonoids [46] that exert neuroprotective activity via inhibiting apoptosis induced by neurotoxins, enhancing memory and learning and promoting cerebrovascular system [47]. Also, anti-inflammatory activities of *ferula* have been confirmed [48].

#### 2. Citrus medica

*C. medica* or citron is called “*Otroj*” in PM, and its peel is one of the important remedies used for enhancing the brain and elimination of fatigue. Fibroblast growth factor-2 (FGF-2) is one of the substances that can induce the growth of neuronal and glial cells. In one study water extract of *C.s medica* could activate FGF-2 promoter in transgenic models [49]. Lowering of reactive oxygen metabolites-derived compounds were observed in rats after supplementation with Citron [50]. The main flavonoids of Citron peel especially hesperetin can traverse from the blood-brain barrier and have no or little adverse effects on normal cells [49]. Hesperetin exhibits neuroprotective effects via multiple mechanisms including antioxidant and anti-inflammatory activities.

#### 3. Asarum europaeum

*A. europaeum* or Asarone that is called “*Asaroon”* in PM is assumed as a neuroprotective plant, and its rhizome is used for fatigue in PM. Asarone is an active compound, which is derived from the rhizome and has two isomers: alpha and beta [51, 52]. β-asarone can cross the blood-brain barrier easily and has significant effects on CNS, while α-asarone can exhibit neuroprotective and antiepileptic activities. Oral treatment with α-asarone in rats after intrahippocampal injection of the amyloid beta peptide (Abeta) could reverse Abeta toxicity and neuronal apoptosis induced by it [53]. Moreover, anti-inflammatory actions have been demonstrated for both α-asarone [54] and β-asarone[55].

#### 4. Ficus carica

*F. carica* or fig called “*Anjir*” in PM has been broadly used as a brain tonic and for treatment of fatigue. Fig has antioxidant properties that are correlated with the number of polyphenols and anthocyanin content of it [56]. Acetylcholinesterase (AChE) and butyrylcholinesterase (BChE) inhibiting activities of fig were demonstrated in vitro [57]. Cholinesterase inhibition can modulate glial cells activation, changes in cerebral blood flow, phosphorylation of tau proteins and the amyloid peptides cascade [58]. In a study on transgenic mice, the group that fed with a supplementary diet with 4% fig, showed significantly lower AChE activity in their hippocampus and cerebral cortex as well as higher antioxidant enzyme activities [59]. Fig extract has increased the level of serotonin and norepinephrine and showed anxiolytic activity in mice [60]. Moreover, the anti-inflammatory effects of fig leaves have been documented in some studies [61, 62].

#### 5. Juglans Regia

*J. regia* or walnut is broadly used in PM as norotonic for promoting mind and alleviation of fatigue. Pretreatment of rat hippocampal cells with walnut extract could significantly increase the viability of them against dopamine or lipopolysaccharide. Walnut extract helped even the cells to obtain their baseline calcium levels after depolarization [63]. Anti-inflammatory activities have been reported from both walnut leaves [64] and fruits, attributed to one of its main constituents, Ellagic acid. Summary of the activities of these plants is exhibited in Table-2.

## Discussion

MS is a common neurologic disease that most of its sufferers complain of fatigue. Despite many studies done to clear the pathophysiology of fatigue in MS, the exact mechanisms and factors have not been fully understood. As it has been explained in detail, recent studies have indicated that fatigue in MS has mostly a central origin. Multiple mechanisms are suggested for fatigue in MS including inflammatory processes and cytokines effects, changes in endocrine systems, axonal loss and changes in cerebral activation paths [18] and alterations in brain neurotransmitters especially serotonin and dopamine [24, 65]. Atrophies in some parts of the brain mainly frontal lobes and basal ganglia have seen in brain imaging from fatigued MS patients. It is probable that weakness or disruption in some neuronal pathways of the brain due to demyelination or axonal loss result in early fatigue with little exertion. All of these changes can be summarized as the term brain dysfunction. [Braley, 2010 #46]PM literatures explain that fatigue (*I’ya*) can be the outcome of exertion or not. The latter (spontaneous *I’ya*) may be prodromal of a disease and can be seen in many chronic diseases especially neurologic illnesses. As we know, fatigue may precede a new relapse of MS and often is accompanying the relapses [66]. Considering the high prevalence of fatigue in MS patients (almost the highest among the neurologic diseases), MS fatigue can be adopted as one of the best equivalents to spontaneous *I’ya*. As a result, the proposed remedies for *I’ya* in PM literatures may benefit MS-fatigue. *Ibn Roshd* has introduced the weakness of the essential organs such as the brain as one of the etiologies of spontaneous *I’ya.* It is reasonable to consider brain atrophy or dysfunction as brain weakness. Due to the role of brain dysfunction or atrophy in developing fatigue in MS patients, neurotonic agents may be useful in resolving fatigue in MS patients by brain reinforcement. In this article, we mentioned some of the herbs that have been used traditionally for brain reinforcement and have showed documented effects in phytotherapic studies (in vitro, in vivo or in cilico). *F. assa-foetida* oleo-gum-resin (*Heltit*), *C. medica* (*Otroj*), *A. europaeum* (*Asaroon*)*, F. carica* or fig (*Anjir*) and *J. regia* or walnut are some examples of neuroprotective drugs used in PM for treatment of fatigue, but there are so many other herbs mentioned in PM texts as neurotonic and brain enhancer. The role of oxidative stress in inducing fatigue in chronic fatigue syndrome and the efficacy of antioxidants in relieving fatigue have been demonstrated [67];so antioxidant activities of the plants indicated above, can play a crucial role in reducing fatigue. As mentioned before, one of the probable etiologies of fatigue in MS is inflammation in the CNS; moreover, as explained separately about each of these neurotonic plants, all of them can suppress inflammation and fight against fatigue via this mechanism. Other mechanisms such as cholinergic activities may play a role in the anti-fatigue activity of these plants that can enhance memory and reduce mental fatigue.

The drugs that are empirically been used nowadays for reducing MS fatigue may act through influencing on one or more of the mentioned paths; for example, it is speculated that Amantadine acts via its immune-mediated effects, amphetamine-like activity or even antiviral actions [68]. Therefore, considering the characteristics of the mentioned plants in this article, they can be used for developing new drugs for MS fatigue.

## Conclusion

The term “*I’ya*” in PM old texts has been used for pathologic fatigue. The spontaneous *I’ya* can be considered equivalent to fatigue due to MS. Some herbs have been introduced in PM literature for reducing fatigue. These medicinal plants can be considered a valuable and noteworthy source for discovering new drugs for MS fatigue. For obtaining more conclusive results and evaluating the effects of above-mentioned medicinal herbs in fatigue, we recommend preclinical and clinical studies.

## Acknowledgment

We appreciate Dr. Fahimeh Habibi and Dr. Fatemeh Alizadeh for editing and English revision of this article.

## Conflicts of Interest

The authors have no conflicts of interest in writing this article.

**Table 1:** Evidences for Role of Basal Ganglia and Cortex in Fatigue due to MS

**Study**	**Modality used**	**Questionnaires used**	**Evidence for involvement in**			
			**BG‡**	**frontal cortex**	**Parietal cortex**	**Temporal cortex**
Pellicano *et al*. 2010	MRI*	MFIS†			Posterior parietal cortex atrophy	
Derache *et al*. 2013	MRI PET^§^	EMIF-SEP^||^	↓density of GM^¶^	↓density of GM	↓density of GM	↓density of GM
Riccitelli *et al*. 2011	MRI			Left precentral GM atrophy		
Téllez *et al*. 2008	MRS**	MFIS, FSS^††^	↓NAA/Cr^‡‡^	No difference		
Wilting *et al*. 2015	MRI		Morphologic &microstructural changes in thalamus			
Rocca *et al*. 2016	fMRI^§§^	MFIS	↓Recruitment	↑activity in Right middle frontal		
Calabrese [69] *et al*. 2010		MFIS, FSS	↓density of GM	↓density of GM	↓density of GM	
Deluca *et al*. 2008	fMRI		Increased activity	Increased activity	Increased activity	
Roelcke *et al*. 1997	PET	FSS	↓cerebral glucose metabolism	↓cerebral glucose metabolism (reverse relation with FSS)		
Colombo *et al*. 2000	MRI	FSS			↑lesion load	
Leocani *et al*. 2001	EEG^¶¶^		↓activity of inhibitory circuits after termination of a simple motor task (cortical overactivity in motor planning) in fatigued MS patients
Tartaglia *et al*. 2004	MRS	FSS	↓NAA/Cr
Sepulcre *et al*. 2009	MRI	MFIS		GM atrophy/ Left frontal WM*** lesion load	Right parietotemporal WM lesion load
Morgante *et al*. 2011	MRI			↑lesion load Only in frontal lobe	
	TMS^†††^			Lack of pre-movement facilitation with rTMS in fatigued MS

*Magnetic Resonance Imaging; †Modified Fatigue Impact Scale; ‡Basal ganglia; §Positron emission tomography; ||a validated self-report questionnaire in French; ¶Gray matter; ** magnetic resonance spectroscopy: ††Fatigue severity scale; ‡‡N-acetyl aspartate/creatine; §§Functional magnetic resonance imaging; ¶¶Electroencephalography; ***White matter; †††Transcranial magnetic stimulation

**Table 2:** Medicinal Plants for Fatigue According to PM

**Scientific name of the herb**	**Anti inflammatory activity**	**Neuroprotection via anti-oxidant activity**	**Other neuroprotective activities**
Ferula-assa foetida	+	-Reducing glutamate induced cell death -Inhibiting ICAM-1* expression	FA can stimulate nerve regeneration
Citrus Medica	+	Hesperetin lowers reactive oxygen metabolites-derived compounds	activate FGF-2† promoter
Asarum europaeum	+	Reversing Abeta toxicity and neuronal apoptosis	
Ficus Carica	+ (fig leaves)	Polyphenols and anthocyanin as anti-oxidants	-Cholinestrase inhibition -lowering beta amyloid
Juglans Regia	+ (ellagic acid)	Protection against dopamine or lipopolysaccharide	

*Intercellular adhesion molecule – 1 † Fibroblast Growth Factor-2
